# *BRCA1 and BRCA2* mutation spectrum – an update on mutation distribution in a large cancer genetics clinic in Norway

**DOI:** 10.1186/s13053-017-0085-6

**Published:** 2018-01-10

**Authors:** Cecilie Heramb, Teresia Wangensteen, Eli Marie Grindedal, Sarah Louise Ariansen, Sheba Lothe, Ketil Riddervold Heimdal, Lovise Mæhle

**Affiliations:** 1 Department of Medical Genetics, Oslo University Hospital, Oslo, Norway; 2 University of Oslo, Oslo, Norway; 3 Norwegian National Advisory Unit on Women’s Health, Oslo University Hospital, Oslo, Norway; 4 Department of Medical Genetics, MSc Oslo University Hospital, Oslo, Norway

**Keywords:** *BRCA1*, *BRCA2*, Founder mutations, Genetic testing

## Abstract

**Background:**

Founder mutations in the two breast cancer genes, *BRCA1* and *BRCA2*, have been described in many populations, among these are Ashkenazi-Jewish, Polish, Norwegian and Icelandic. Founder mutation testing in patients with relevant ancestry has been a cost-efficient approach in such populations. Four Norwegian *BRCA1* founder mutations were defined by haplotyping in 2001, and accounted for 68% of *BRCA1* mutation carriers at the time. After 15 more years of genetic testing, updated knowledge on the mutation spectrum of both *BRCA1* and *BRCA2* in Norway is needed. In this study, we aim at describing the mutation spectrum and frequencies in the *BRCA1/2* carrier population of the largest clinic of hereditary cancer in Norway.

**Methods:**

A total of 2430 *BRCA1* carriers from 669 different families, and 1092 *BRCA2* carriers from 312 different families were included in a quality of care study. All variants were evaluated regarding pathogenicity following ACMG/ENIGMA criteria. The variants were assessed in AlaMut and supplementary databases to determine whether they were known to be founder mutations in other populations.

**Results:**

There were 120 different *BRCA1* and 87 different *BRCA2* variants among the mutation carriers. Forty-six per cent of the registered *BRCA1/2* families (454/981) had a previously reported Norwegian founder mutation. The majority of *BRCA1/2* mutations (71%) were rare, each found in only one or two families. Fifteen per cent of *BRCA1* families and 25% of *BRCA2* families had one of these rare variants. The four well-known Norwegian *BRCA1* founder mutations previously confirmed through haplotyping were still the four most frequent mutations in *BRCA1* carriers, but the proportion of *BRCA1* mutation carriers accounted for by these mutations had fallen from 68 to 52%, and hence the founder effect was weaker than previously described.

**Conclusions:**

The spectrum of *BRCA1* and *BRCA2* mutations in the carrier population at Norway’s largest cancer genetics clinic is diverse, and with a weaker founder effect than previously described. As a consequence, retesting the families that previously have been tested with specific tests/founder mutation tests should be a prioritised strategy to find more mutation positive families and possibly prevent cancer in healthy relatives.

## Background

Breast cancer genes 1 and 2, *BRCA1/2* have been very well studied since their discovery in 1994 and 1995. Disease-causing mutations in these genes give a high lifetime risk of both breast and ovarian cancer [1–3]. An increased risk of aggressive prostate cancer for male *BRCA2* mutation carriers has been described [4], as well as elevated risk of pancreatic cancer [5]. Risk for other cancers is less evident [5, 6]. Preventive measures such as prophylactic mastectomy and oophorectomy or surveillance with breast MRI seem to improve survival for *BRCA* mutation carriers without significantly reducing quality of life [7–10]. More recently also cancer treatment choices are influenced by *BRCA*-status, especially for ovarian cancer [11].

Founder mutations in *BRCA1* and *2* have been described in many populations, i.e. the Ashkenazi-Jewish, Polish, Norwegian, and Icelandic [12–14]. Therefore, founder mutation testing in patients with relevant ancestry and family history has been a cost-efficient approach during the years with limited access to sequence analysis. A founder mutation may be defined as “a genetic alteration observed with high frequency in a group that is or was geographically or culturally isolated, in which one or more of the ancestors was a carrier of the altered gene”. Founder effect is frequently defined as “the loss of genetic variation that occurs when a new population is established by a very small number of individuals from a larger population” [15]. Different historical, societal and geographic factors may influence development of a founder effect including bottle neck phenomenon, genetic drift, selective mating /inbreeding, and high reproduction.

One of the first studies carried out on *BRCA* epidemiology in Norway by Moller et al. in 2001, showed that 68% of the mutation carriers had one of the four most frequent Norwegian founder mutations in *BRCA1* [16], c.1016dup, c.1556del, c.3328_3229del, c.697_698del, all demonstrated to be true founder mutations through haplotyping [13]. The variant c.1016dup was shown to originate in the south-eastern part of the country, while the other three originated from the south-western part of the country, before the Bubonic plague. Later, in 2007, four more *BRCA1* variants and two *BRCA2* variants c.3847_3848del and c.2808_2811del were published as frequent mutations in the Norwegian population, but no haplotype study has been carried out to establish a true founder origin in these [12, 17].

Founder mutation testing and MLPA (multiplex ligation-dependent probe amplification) have a lower sensitivity compared to sequencing of the entire genes and MLPA especially when used on a population with mixed genetic background. This has become increasingly obvious in our clinical practice over the years. Due to the multicultural population served by Oslo University Hospital (OUH), as well as the falling costs of testing, sequencing and MLPA as initial test has been chosen over founder mutation testing when *BRCA*-testing is indicated. Following this, sequencing and MLPA have become the standard test since January 2014. This practice is in line with the fact that genetic variation in any gene is abundant, and rare, pathogenic variants in any gene are expected to exist [18].

The knowledge on frequencies and spectrum of disease-causing variation in *BRCA1/2* both nationally and locally is however incomplete. The aim of this study has been to describe the results from the *BRCA* testing during the last 15 to 20 years. This will give necessary overview of mutation frequencies in our region, and the results may give directions for both future research and serve as an evaluation of the current testing practice.

## Methods

### Study design

The study was carried out in the Section of Hereditary Cancer, Department of Medical Genetics, Oslo University Hospital, OUH, and was approved by the Data Protection Officer at OUH as a quality of care study. The study group was the full mutation carrier population registered in the clinic. Data collection was done in May 2016, and mutation carriers registered in the clinic before 5th of May 2016 were included. The study subjects were both men and women tested over the years, affected with cancer or not. Families registered with a positive mutation test were included. The lowest number of mutation carriers in a family was set to one. In this study, “family” was defined by the practice of giving an index patient a separate family number if he/she did not already have family members registered in the clinic. A thorough job looking up relatives have been done in each case and if relatives were found, the person have been included in the already registered family. Genetic testing was performed both diagnostically and predictively. All activities fulfilled the requirements of genetic counselling, information and consent stated by the Norwegian Act on Biotechnology, www.lovdata.no. All clinical information was registered in the electronic patient journals at OUH. Close to all positive mutation tests were confirmed in a separate blood sample. On the basis of the selection criteria, 2430 *BRCA1* mutation carriers from 669 different families, and 1092 *BRCA2* carriers from 312 different families were included in the study.

### Genetic testing and testing strategies

Our cancer genetics clinic has offered both diagnostic and predictive testing to individuals fulfilling criteria for *BRCA1/2* testing given by the health authorities. Initially our clinic served the whole country with genetic counselling and *BRCA* testing. Since the late 90s, the department has mainly served the South-Eastern part of Norway. The south-eastern part of Norway contains 2.9 million people, which is a little more than half of the Norwegian population of 5.2 mill.

From around 1995 and onwards, the laboratories performing *BRCA* analysis used various techniques. Initially, by using techniques such as denaturing gradient gel electrophoresis and sequencing methods, four recurrent *BRCA1* mutations were identified in Norwegian families (c.1556del, c.3328_3229del, c.697_698del and c.1016dup) [13]. Eventually other cost-efficient/affordable tests, such as multiplex PCR fragment analysis and sequencing of shorter fragments were used to screen larger groups of individuals, as well as to detect mutations already found in the family. When new frequent mutations were identified these were included in the fragment analysis tests.

Sequencing of *BRCA1* and *2* genes has increasingly been offered to our high-risk cancer families since 2000 and 2002 respectively, and MLPA analysis since 2002. Fragment analysis and sequencing/MLPA were used interchangeably in the work-up of these patients until January 2014, when Sanger or high throughput sequencing (HTS) methods have been used combined with MLPA. It should be noted that patients from families with a known genetic mutation have only been tested for this specific mutation except when more than one mutation is suspected. This applied to fourteen families where two mutations in *BRCA1 /BRCA2* were identified.

### Founder mutation search method

To establish whether the variants found in our cohort were described as founder mutations elsewhere, we used the following strategy: All variants were described with HGVS standard nomenclature (*BRCA1* NM_007294.3 and *BRCA2* NM_000059.3). A search was performed in Alamut Visual per variant, first with default settings, then adding “founder” to the variant search terms. Alamut searches automatically after all known notations of the variant, mainly in Google. Depending on the search results, the variants were termed either F = Founder, when documentation of this was retrieved, NF = Not founder, when the variant was previously reported but not shown to be a founder anywhere, or NPR = Not previously reported if there were no documents retrievable on the variant. A double check on all variants initially classified as NPR was performed in databases ClinVar, HGMD, UMD, LOVD and *BRCA* Exchange.

### Classification

The original laboratory reports were from different time periods with different routines for variant interpretation and reporting. In order to ensure up to date quality of the variant classification, we reevaluated all mutations reported in the *BRCA1/2* carrier population as part of this quality of care study. Variants were interpreted according to the recommendations of the American College of Medical Genetics [19], and ENIGMA (Evidence-based Network for the Interpretation of Germline Mutant Alleles) using the five-class system: pathogenic (class 5), likely pathogenic (class 4), variant of uncertain significance (class 3), likely benign (class 2), or benign (class 1). A disease-causing mutation was defined as a class 4 or 5 variant according to ACMG/ ENIGMA criteria. The types of mutations for both genes are listed in Table 1, all classified as 4 or 5. The majority of variants were straight forward to classify as they introduce stop or frameshift, or constitute rearrangements or alter splicing. The splicing mutations were either in canonical +/−1 or 2 splice sites or analyzed in functional test by us or others. The missense mutations were identified in the well-known domains, RING and BRCT in *BRCA1* and DNA binding domain in *BRCA2*. Published multifactorial likelihood scores and/ or functional studies were part of the evidence in these cases. Disease-causing missense mutations were found to constitute 9% of *BRCA1* and 5% of *BRCA2* mutations in this study. Others have suggested that approximately 7% of the load of pathogenic sequence variants in *BRCA1* is attributable to missense substitutions [20, 21]. Variants of unknown significance (VUS) were not included in this study.

**Table 1:** Types of mutations

Type of mutation	*BRCA1*	*BRCA2*
Frame shift	49	45
Stop	38	25
Rearrangement	12	4
Missense	11	4
Splice variant	9	7
Start loss	1	1
In frame deletion	0	1
	120	87

For the purpose of this study, a *founder mutation* was defined as a variant previously reported as such, and this may include common ancestry proven by haplotype studies. A *recurrent* mutation was defined as a variant to occur in one mutational hot spot as separate events, this term is however used synonymously with frequent variant in many publications. In this study, a *frequent mutation* was defined as a mutation found in three or more families and subdivided into three categories for systematic purposes. Mutations found in >30 different families each were termed *highly frequent*, mutations found in 10–30 families were termed *moderately frequent*. Mutations found in 3–9 families each were termed *less frequent*. A *rare mutation* was defined as a mutation found in one or two families. A frequent mutation from any of the three groups may in some cases be considered a founder candidate, depending on e.g. the geographical origin of the families. Any mutation, both frequent and rare in our study, may be known as founder mutations in a specific population.

### Mutation frequencies

Throughout this study we have calculated mutation frequencies both as number of mutation carriers per variant, and number of different families per variant. The fraction of mutation carriers carrying the four well-known *BRCA1* founder mutations are directly comparable to the numbers obtained in the previous studies done on the subject. The number of families per variant would be indicative of possible new founder mutations, which again may be of separate interest for future studies. A calculation of number of mutation carriers per family was included in the work-up for each variant. Establishing a carrier frequency for the population on the whole was beyond the scope of this study.

## Results

**Fig. 1:**
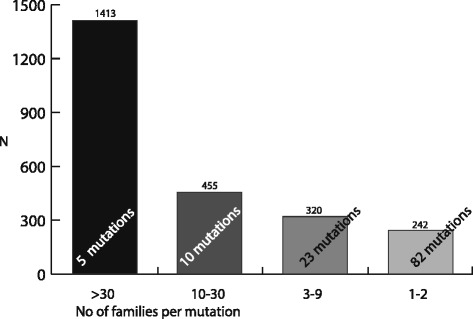
Proportions of *BRCA1* mutation carriers vs frequency of mutations (*N* = 2432)

**Fig. 2:**
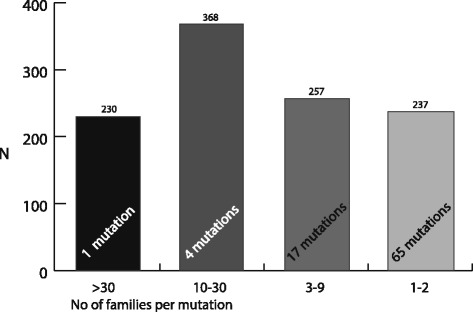
Proportions of *BRCA2* mutation carriers vs frequency of mutations (*N* = 1092)

The *BRCA1* results are shown in Fig. 1 and Table 2, the *BRCA2* results are shown in Fig. 2 and Table 3. There were 120 different *BRCA1* variants and 87 different *BRCA2* variants found among the mutation carriers, 669/981 families had a *BRCA1* mutation (68%), and 312/981 had a *BRCA2* mutation (32%). Forty-six per cent of the registered *BRCA1/2* families (454/981) had a previously known Norwegian founder mutations, identified through the founder search in Alamut. There were five *BRCA1* variants and one *BRCA2* variant among the six most frequent *BRCA1/2* variants (Table 4). These six variants accounted for 47% (1643/3522) of the mutation carriers. In total, 70 % of *BRCA1/2* mutation carriers (2466/3522) had a moderately or highly frequent variant (found in more than 10 families). Sixteen per cent (577/3522) had a less frequent variant found in 3–9 families. Fourteen per cent of *BRCA1/2* carriers (479/3522) had a rare mutation.

**Table 2:** BRCA1 variants

No of families	HGVS		Type of mutation	No.of ind.	No. of fam.	Average no of carr./fam	Percentage of carriers/*families*	Previous reports*
>30	c.1016dup	p.Val340Glyfs*6	fs	471	111	4.2	58%	Norwegian, Danish and Swedish
	c.1556del	p.Lys519Argfs*13	fs	399	95		1413/2430	Norwegian
	c.3228_3229del	p.Gly1077Alafs*8	fs	214	45			Italian, Norwegian
	c.697_698del	p.Val233Asnfs*4	fs	182	44		*51%*	Norwegian
	c.3178G > T	p.Glu1060*	stop	147	46		*341/669*	Norwegian
10–30	c.1A > G	p.Met1Val	start codon	69	21	3.5	19%	Norwegian
	c.3048_3052dup	p.Asn1018Metfs	fs	44	16		455/2430	Swedish founder
	c.5266dup	p.Gln1756Profs*74	fs	30	16			European, Russian
	c.3084_3094del	p.Asn1029Argfs*5	fs	43	13			Norwegian
	dup exon 13/c.(4185 + 1_41861)_(4357 + 1_4358–1)dup	p.?	rearr	41	11		*19%*	Norwegian and British
	dup exon 22 /c.(5332 + 1_5333–1)_(5406 + 1_5407–1)del	p.?	rearr	29	11		*128/669*	Dutch
	c.4745del	p.Asp1582Alafs*19	fs	78	10			Norwegian
	c.2351_2357del	p.Ser784Trpfs*6	fs	54	10			Norwegian
	del exon 8–13 / c.(441 + 1_442–1)_(4357 + 1_4358–1)del	p.?	rearr	37	10			British, European founder
	c.3607C > T	p.Arg1203*	stop	30	10			Greek founder, Belgian, Korean recurrent
3–9	c.1072del	p.Leu358Cysfs*16	fs	22	8	3.2	13%	NF
	c.68_69del	p.Glu23Valfs*17	fs	13	7		320/2430	Ashkenazi, Polish, Italian, Spanish
	c.4065_4068del	p.Asn1355Lysfs*10	fs	12	7			British and German
	c.5047G > T	p.Glu1683*	stop	39	6			NF
	c.3319G > T	p.Glu1107*	stop	16	8		*15%*	Danish
	c.5075-2A > C	p.?	splice var	39	5		*101/669*	Norwegian
	c.2475delC	p.Asp825Glufs*21	fs	15	5			Swedish and Danish
	c.3700_3704del	p.Val1234Glnfs*8	fs	7	5			Rec. Greek, Czech
	c.3331_3334del	p.Gln1111Asnfs*5	fs	21	4			Hispanic, Portuguese founder
	c.2591C > G	p.Ser864*	stop	16	4			NF
	c.3966delA	p.Lys1322Asnfs*3	fs	15	4			NF
	del exon 3–16/ c.(80 + 1_81–1)_(4986 + 1_4987–1)del	p.?	rearr	14	4			NF
	c.130 T > A	p.Cys44Ser	missense	8	4			NF
	c.1450G > T	p.Gly484*	stop	18	3			NF
	c.5513 T > G	p.Val1838Gly	missense	13	3			NF
	c.3756_3759del	p.Ser1253Argfs*10	fs	10	3			Recurrent Polish
	c.5251C > T	p.Arg1751*	stop	10	3			Finnish
	c.1687C > T	p.Gln563*	stop	6	3			European, Austrian, Slovanian founder
	c.3710del	p.Ile1237Asnfs*27	fs	6	3			Danish, Swedish, rec. Polish
	c.4035del	p.Glu1346Lysfs*20	fs	6	3			Slovenian, Polish, Latvian, Lithuanian
	c.5309G > T	p.Gly1770Val	missense	6	3			Moroccan founder
	c.181 T > G	p.Cys61Gly	missense	5	3			Central and eastern European founder
	c.843_846del	p.Ser282Tyrfs*15	fs	3	3			NF
1–2	c.5511G > A	p.Trp1837*	stop	18	2	2.4	10%	NF
	c.2869C > T	p.Gln957*	stop	14	2		242/2430	NF (?)
	c.66dupA	p.Glu23Argfs*18	fs	12	2			NF
	c.1058G > A	p.Trp353*	stop	10	2		*15%*	NF
	c.5407-25 T > A	p.?	splice var	9	2		*99/669*	NF
	c.1292dup	p.Leu431Phefs*5	fs	8	2			NF
	c.794_795del	p.Ser265Cysfs*21	fs	8	2			NF
	c.2558ins356	p.?	stop	*7*	*2*			NPR
	c.5503C > T	p.Arg1835*	stop	6	2			Pakistani founder
	c.2681_2682del	p.Lys894Thrfs*8	fs	5	2			Scottish
	c.2989_2990dup	p.Asn997Lysfs*4	fs	5	2			NF
	c.4689C > G	p.Tyr1563*	stop	5	2			NF
	c.5153G > C	p.Trp1718Ser	missense	5	2			NF
	del exon 1–3 / c.(?_1–1)_(134 + 1_135–1)del	p.?	rearr	4	2			Norwegian founder
	c.1287del	p.Ile429Metfs*12	fs	2	2			NPR
	c.3937C > T	p.Gln1313*	stop	1	1			NF
	c.5095C > T	p.Arg1699Trp	missense	1	2			NF
	c.3874del	p.Ser1292Leufs*15	fs	7	1			Danish founder
	del exon 1–13/ c.(?_1–1)_(4357 + 1_4358–1)del	p.?	rearr	5	1			Norwegian founder
	c.5534del	p.Glu1346Lysfs*20	fs	5	1			NPR
	c.1793 T > G	p.Leu598*	stop	4	1			NF
	c.115 T > G	p.Cys39Gly	missense	2	1			Greenlandic/Danish founder
	del exon 5–7/c.(134 + 1_135–1)_(441 + 1_442–1)del	p.?	rearr	3	1			NF
	del exon 8 c.(441 + 1_442–1)_(547 + 1_548–1)del	p.?	rearr	3	1			European founder
	c.848 T > A	p.Leu283*	stop	3	1		NF	
	c.929del	p.Gln310Argfs*4	fs	3	1			NF
	c.1434_1435del	p.Glu479Lysfs*10	fs	3	1			NF
	c.2257dup	p.Ser753Lysfs*9	fs	3	1			NPR
	c.3770_3771del	p.Glu1257Glyfs*9	fs	3	1			Spanish founder
	c.4612C > T	p.Gln1538*	stop	3	1			NF
	c.457_458ins21	p.?	stop	6	2			NPR
	del exon 18–24/c.(5074 + 1_5075–1)_(5592 + 1_?-1)del	p.?	rearr	2	1			NPR
	c.1059G > A	p.Trp353*	stop	2	1			NF
	c.1360_1361del	p.Ser454*	stop	2	1			Italian
	c.1695dup	p.Lys566Glufs*4	fs	1	1			NF
	c.65 T > C	p.Leu22Ser	missense	2	1			NF
	c.2138C > G	p.Ser713*	stop	1	1			NF
	c.2389G > T	p.Glu797*	stop	2	1			NF
	c.2438dup	p.Leu814Thrfs*9	fs	2	1			NF
	c.3477_3479delinsC	p.Lys1160Glyfs*4	fs	2	1			NF
	c.3689 T > G	p.Leu1230*	stop	2	1			NF
	c.3835del	p.Ala1279Hisfs*28	stop	2	1			NF
	c.4186C > T	p.Gln1396*	stop	2	1			NF
	c.4484G > A	p.Ala1453Glyfs*10	splice var.	2	1			NF
	c.386del	p.Gly129Alafs*34	fs	2	1			NPR
	c.4932_4933dup	p.Arg1645Lysfs*14	fs	2	1			NF
	c.4972delA	p.Thr1658Profs*19	fs	2	1			NPR
	c.4986 + 1G > T	p.?	splice var	2	1			NF
	c.5407-2A > G	p.?	splice var	2	1			NF
	c.445G > T	p.Glu149*	stop	2	1			NPR
	c.510del	p.Ile171Tyrfs*63	fs	2	1			NPR
	del exon 11/c.(670 + 1_671–1)_(4096 + 1_4097–1)del	p.?	rearr	1	1			NPR
	del exon 16/c.(4675 + 1_4676–1)_(4986 + 1_4987–1)del	p.?	rearr	1	1			NF
	del exon 20–24/c.(5193 + 1_5194–1)_(5592 + 1_?-1)del	p.?	rearr	1	1			NF
	c.1175_1214del	p.Leu392Glnfs*5	fs	1	1			NF
	c.1674dup	p.Gly559Argfs*2	fs	1	1			NPR
	c.1823_1826del	p.Lys608Ilefs*3	fs	1	1			NF
	c.1961dup	p.Tyr655Valfs*18	fs	1	1			NF
	c.2019del	p.Glu673Aspfs*28	fs	1	1			NF
	c.2185G > T	p.Glu729*	stop	1	1			NF
	c.2293G > T	p.Glu765*	stop	1	1			NF
	c.140G > T	p.Cys47Phe	missense	1	1			NF
	c.2727_2730del	p.Asn909Lysfs*90	fs	1	1			NF
	c.2864C > A	p.Ser955*	stop	1	1			Hispanic, Californian
	c.188 T > A	p.Leu63*	stop	1	1			Japanese
	c.2981_2982del	p.Cys994*	stop	1	1			NF
	c.3005del	p.Asn1002Thrfs*22	fs	1	1			NF
	c.213-5 T > A	p.?	splice var	1	1			NF
	c.3400G > T	p.Glu1134*	stop	1	1			NF
	c.241C > T	p.Gln81*	stop	1	1			NF
	c.3544C > T	p.Gln1182*	stop	1	1			NF
	c.3644_3648del	p.(Asn1215Ilefs*2)	fs	1	1			NPR
	c.3813dupT	p.(Asn1272*)	stop	1	1			NF
	c.3817C > T	p.Gln1273*	stop	1	1			NF
	c.4146_4155dup	p.Ser1386Leufs*8	fs	1	1			NF
	c.5074 + 2 T > C	p.?	splice var.	1	1			NF
	c.5030_5033del	p.Thr1677Ilefs*2	fs	1	1			French
	c.5212G > A	p.Gly1738Arg	missense	1	1			Greek
	c.5193 + 2del	p.?	splice var	1	1			NF
	c.5434C > G	p.Gly1803Glnfs*11	splice var.	1	1			NF
	c.514C > T	p.Gln172*	stop	1	1			NF
	c.5213G > A	p.Gly1738Glu	missense	1	1			Danish, Iranian

*Founders are reported with indication of origin, *NF* not founder through search, *NPR* not previously reported

**Table 3:** BRCA2 variants

No of families	HGVS		Type of mutation	No.of ind.	No. of fam.	Average no of carrier/ fam	Percentage of carriers*/families*	Previous reports
>30	c.5217_5223del	p.Tyr1739*	stop	230	61	3,8	21%	NF
							230/1092 *19%* *61/312*	
10–30	c.4821_4823delTGAinsC	p.Glu1608Aspfs*6	fs	126	25	3,9	34%	NF
	c.2808_2811del	p.Ala938Profs*21	fs	89	26		368/1092	Norwegian founder
	c.8331 + 2 T > C	p.?	splice variant	71	29			Danish founder
	c.3847_3848del	p.Val1283Lysfs*2	fs	82	14		*30%* *94/312*	Norwegian, Iranian founder
3–9	dup exon 20/ c.(8487 + 1_8488–1)_(8632 + 1_8633–1)dup	p.?	rearr	34	9	3,2	23%	NF
	c.9118-2A > G	p.?	splice variant	44	8		257/1092	Finnish founder, recurrent Polish
	c.5723_5722del	p.Leu1908Argfs*2	fs	23	8			NF
	c.2047_2050del	p.Ser683Argfs*46	fs	28	6		*26%* *80/312*	NPR
	c.8229_8243del	p.Arg2744_Gly2748del	in frame del	18	5			NF
	c.5946del	p.Ser1982Argfs*22	fs	11	5			Ashkenazi, Hungarian
	c.771_775del	p.Asn257Lysfs*17	fs	6	5			Finnish, Icelandic
	c.1905_1909del	Asp635Glufs*15	fs	22	4			NF
	c.5576_5573del	p.Ile1859Lysfs*5	fs	11	4			NF
	c.6059_6062del	p.Glu2020Valfs*19	fs	9	4			NF
	c.9117G > A	p.Val2985Glyfs*4	splice variant	5	4			NF
	c.171C > G	p.Tyr57Ter*	stop	15	3			NF
	c.8177A > G	p.Tyr2726Cys	missense	9	3			NF
	c.2830A > T	p.Lys944Ter*	stop	8	3			NF
	c.3847_3848del	p.Val1283Lysfs*2	fs	8	3			NF
	c.7069_7070del	p.Leu2357Valfs*2	fs	3	3			NF
	c.7480C > T	p.Arg2494*	stop	3	3			Finnish founder
1–2	del exon 3	p.?	rearr	8	2	3,1	22%	NF
	c.3G > A	p.Met1?	start loss	6	2		237/1092	NF
	c.5157_5161del	p.Asn1719Lysfs*6	fs	2	2		*25%*	NF
	c.6373dup	p.Thr2125Asnfs*4	fs	5	2		*77/312*	NF
	c.6486_6489del	p.Lys2162Asnfs*5	fs	2	2			Danish founder
	c.7558C > T	p.Arg2520*	stop	14	2			NF
	c.7617 + 1G > A	p.?	splice	2	2			Danish founder
	c.8130del	p.Ser2710Argfs*23	fs	9	2			NF
	c.8323dup	p.Met2775Asnfs*7	fs	10	2			NF
	c.631 + 4A > G	p.?	splice	11	2			NF
	c.9154C > T	p.Arg3052Trp	missense	9	2			NF
	c.9699_9702del	p.Cys3233Trpfs*15	fs	12	2			NF
	del exon 19–21/c.(8331 + 1_8332–1)_(8754 + 1_8755–1)del	p.?	rearr	1	1			NPR
	del exon 20/c.(8487 + 1_8488–1)_(8632 + 1_8633–1)del	p.?	rearr	1	1			NF
	c.1296_1297del	p.Asn433Glnfs*18	fs	1	1			NF
	c.1429del	p.His477Ilefs*8	fs	10	1			NPR
	c.1456C > T	p.Gln486*	stop	5	1			NF
	c.1642C > T	p.Gln548*	stop	3	1			NF
	c.1658 T > G	p.Leu553*	stop	4	1			NF
	c.1945C > T	p.Gln649*	stop	4	1			NF
	c.2636_2637del	p.Ser879*	stop	2	1			NF
	c.3158 T > G	p.Leu1053*	stop	1	1			NF
	c.3307_3308dup	p-Leu1103Phefs*2	fs	1	1			NPR
	c.3545_3546del	p.Phe1182*	stop	1	1			NF
	c.3596_3599del	p.Asp1199Valfs*9	fs	8	1			NF
	c.3720_3721del	p.Phe1241*	stop	2	1			NPR
	c.3751dup	p.Thr1251Asnfs*14	fs	2	1			NF
	c.171del	p.Tyr57*	stop	4	1			NPR
	c.3860del	p.Asn1287Ilefs*6	fs	1	1			Austrian founder
	c.196C > T	p.Gln66*	stop	2	1			NF
	c.4095 T > A	p.Cys1365*	stop	3	1			NF
	c.4258del	p.Asp1420Ilefs*28	fs	2	1			Swedish founder
	c.4710del	p.GLu1571Argfs*8	fs	1	1			NF
	c.4794_4797del	p.Asn1599Metfs*17	fs	1	1			NF
	c.5073dup	p.Trp1692Metfs*3	fs	1	1			NF
	c.316 + 1G > T	p.?	splice variant	5	1			NF
	c.5577del	p.Val1862*	stop	1	1			NF
	c.5645C > A	p.Ser1882*	stop	7	1			French founder
	c.5682C > G	p.Tyr1894*	stop	4	1			NF
	c.6034dup	p.Ser2012Phefs*6	fs	3	1			NPR
	c.6084_6088del	p.Glu2029Tyrfs*18	fs	5	1			NPR
	c.407del	p.Asn136 Ilefs*16	fs	1	1			NF
	c.6611dup	p.Val2205Cysfs*20	fs	2	1			NF
	c.6591_6592del	p.Glu2198Asnfs*4	fs	3	1			NF
	c.469_470del	p.Lys157Valfs*25	fs	3	1			NF
	c.7024C > T	p.Gln2342*	stop	1	1			NF
	c.517-2A > G	p.?	splice variant	2	1			NF
	c.7234del	p.Thr2412Leufs*57	fs	4	1			NF
	c.7673_7674del	p.Glu2558Valfs*7	fs	3	1			NF
	c.7680dup	p.Gln2561Serfs*5	fs	1	1			NF
	c.7753G > A	p.Gly2585Arg	missense	1	1			NF
	c.7829dupT	p.Asp2611Glyfs*7	fs	1	1			NF
	c.7878G > C	p.Trp2626Cys	missense	3	1			NF
	c.7913_7917del	p.Phe2638*	stop	1	1			Czech founder
	c.8090_8105del	p.Ser2697Lysfs*31	fs	2	1			NPR
	c.8396dup	p.Pro2800Thyfs*12	fs	9	1			NPR
	c.658_659del	p.Val220Ilefs*4	fs	1	1			Lithuanian founder
	c.8878C > T	p.Gln2960*	stop	1	1			Korean
	c.614delG	p.Ser205Ilefs*6	fs	4	1			NPR
	c.9127G > T	p.Glu3043*	stop	1	1			NF
	c.9227del	p.Gly3076Aspfs*7	fs	2	1			NF
	c.9253dup	p.Thr3085Asnfs*26	fs	10	1			NF
	c.9382C > T	p.Arg3128*	stop	1	1			Jewish founder
	c.9403del	p.Leu3135Phefs*28	fs	1	1			NF
	c.9523G > T	p.Glu3175*	stop	3	1			NF

*Founders are reported with indication of origin, *NF* not founder through search, *NPR* not previously reported

**Table 4:** *BRCA1/2* variants found in more than 30 families

Gene	Variant		No. of individuals	No. of families
*BRCA1*	c.1016dup	p.Val340Glyfs*6	471	111
*BRCA1*	c.1556del	p.Lys519Argfs*13	399	95
*BRCA2*	c.5217_5223del	p.Tyr1739*	230	61
*BRCA1*	c.3178G > T	p.Glu1060*	147	46
*BRCA1*	c.3228_3229del	p.Gly1077Alafs*8	214	45
*BRCA1*	c.697_698del	p.Val233Asnfs*4	182	44
Total			1643	403

### BRCA1

Each of the four well-known founder mutations in *BRCA1*, c.1556del, c.3328_3229del, c.697_698del and c.1016dup, were found in more than 30 different families each and classified as highly frequent (Table 2, Fig. 1). These four mutations accounted for 52% (1266/2430) of *BRCA1* carriers in this study, or 44% of *BRCA1* families (295/669). The variant c.1016dup was the most frequent mutation with 471 mutation carriers from 111 families. Together with the fifth highly frequent mutation, c.3178 G > T, also found in more than 30 families, the top five *BRCA1* mutations accounted for 58% (1413/2430) of the mutation carriers, or 51% of the families (341/669).

Twenty-seven per cent (33/120) of the variants were classified as moderately frequent (10 variants) and less frequent (23 variants). These accounted for 32% of mutation carriers (775/2430) or 34% of *BRCA1* mutation families (229/669).

Sixty-eight per cent (82/120) of the *BRCA1* variants were rare. Ten per cent of the *BRCA1* mutation carriers (242/2430), 15% of the *BRCA1* families (99/669), had one of these mutations.

### BRCA2

The single most frequent *BRCA2* variant, c.5217_5223del, was found in 230 individuals from 61 different families. This variant accounted for 21% (230 /1092) of *BRCA2* carriers, or 19% of families (61/312). It was also the third most prevalent variant when both *BRCA1/2* were taken together (Table 4), but it was not found to be reported as a founder from the Alamut search.

Four moderately frequent mutations were found, (c.4821_4823delTGAinsC, c.2808_2811del, c.8331 + 2 T > C and c.3847_3848del), and they accounted for 34% (368/1092) of the *BRCA2* mutation carriers, or 30% (94/312) of the families.

Twenty per cent (17/87) of the *BRCA2* variants were classified as less frequent, accounting for 23% (257/1092) of the mutation carriers, or 26% (80/312) of the families.

Seventy-five per cent (65/87) of the *BRCA2* variants were considered rare, found in 1–2 families each. Twenty-two per cent (237/1092) of the mutation carriers, (25% (77/312) of the families) had one of these rare *BRCA2* mutations.

### Founder mutation search

Among the variants found in more than ten families each, ten out of fifteen *BRCA1* variants and two out of five *BRCA2* variants were previously reported as founder mutations in Norway, including the four demonstrated by haplotyping. Another three *BRCA1* variants were reported as Norwegian founders, and these were found in the less frequent or rare category. The remaining highly frequent variants were either described as founder mutations in neighbouring/European countries, or previously reported in other countries, but not as founders, which was the case with the two most frequent *BRCA2* variants (c.5217_5223del and c.4821_4823delTGAinsC). There were 14 founder mutations among the 23 less frequent mutations in *BRCA1* (61%), mainly Central-European, one Norwegian and three Swedish/Danish. There were four founder mutations among the 17 less frequent *BRCA2* variants (23.5%), none of them were Norwegian*.* Seventeen per cent of the rare *BRCA1* variants (14/82) and 15.4% of *BRCA2* variants (10/65) were previously described as founders. Details on founder mutation origin are listed in Tables 2 and 3.

Variants not previously reported (NPR) were found mainly among the rare variants for both genes. Thirteen *BRCA1* and 10 *BRCA2* variants (8.2 and 11.5% of variants, respectively) were not previously described in the Alamut search or in available databases. The *BRCA2* variant c.2047_2050del, found in six families, was the only frequent variant not previously described.

## Discussion

We aimed at describing the *BRCA1/2* mutation distribution in the largest genetic clinic in Norway after many years of *BRCA* testing. Over the last 10 years, the total number of mutation carriers (*N* = 3522) is almost 2.5-fold and the total number of mutations has almost tripled (*N* = 207), compared to 2007 when 1300 carriers and 75 distinct mutations were identified [17].

There are three main findings. Firstly, the distribution of both *BRCA1* and *BRCA2* mutations is quite extreme: A few mutations are very frequent and many mutations are very rare. The four proven *BRCA1* founder mutations by Møller et al. in 2001 are still the most common variants among *BRCA1* mutation carriers, but these variants account for a smaller proportion of carriers than previously described. Secondly, there is an increasing amount of moderately and less frequent variants in both genes, among which many are considered to be founders. This is especially true for the *BRCA2* variant c.5217_522del which is not previously described as a founder, but is shown to be the third most frequent mutation in the *BRCA1/2* carrier population as a whole. Thirdly, 71% (148/208) of the *BRCA1/2* variants are rare and found in only one or two individuals/families.

Even though the four most common *BRCA1* mutations are the exact same in 2016 as in 2001 [13], the proportion of *BRCA1* mutation carriers accounted for by the four founders has fallen from 68% in 2001 to 52% in the present study. In the 2001- study, 82 patients who contracted breast cancer prospectively after being recommended breast cancer screening based on their family history, were *BRCA1/2* tested. No *BRCA2*- mutation were found. The patients were included for breast cancer screening based on selection criteria similar to traditional testing criteria. The present study has a retrospective method, and a much higher number of patients compared to the previous study, and any rare mutation will be easier to detect.

As expected when testing more patients, some of the rare/less frequent variants described in 2001 are shown to be frequent, as is the case with the *BRCA1* variant c.3178G > T [16]. On the other hand, the *BRCA1* variants c.794_795del, c.2558ins356, c.2869C > T and c.5511G > A were all identified in 2001, and have not turned out to be frequent in the patient population over time. It remains to be seen from future testing which of the rare variants in 2016 that remains rare.

The second main finding is that a substantial number of carriers have moderately or less frequent mutations, many of which are founder candidates. The laboratories have, as specified in Method section, offered specific testing for the frequent mutations that have been detected over time. Finding a mutation in more than three families is a liberal but well recognized threshold of suspecting a founder candidate [12, 22]. Both the well-known Icelandic/Finnish founder, *BRCA2* c.771_775del, some of the European recurrent mutations, i.e. *BRCA1* c.5266dup and, c.181 T > G as well the Ashkenazi Jewish founders are all present in the groups of low or moderately frequent mutations in our study. A recently identified Moroccan *BRCA1* variant has been demonstrated in three families at OUH and have been shown to share the same haplotype as in a series of Moroccan patients [23]. Systematic collection of information on geographic or ethnic origin per individual/family was beyond the scope and permits of this study, but was performed in 2007, where it was found to be an apparent geographical connection for some of the frequent mutations in *BRCA1* and *BRCA2* [17]*.*

The third important finding is that 71% of the *BRCA1/2* variants are classified as rare, and that 14% of the mutation carriers in total have one of these rare variants, 10% of *BRCA1* carriers and 22% of *BRCA2* carriers. Some of these variants are actually reported as founders, mainly from other populations (15 and 17% of variants for *BRCA1* and *BRCA2* respectively), and some are not previously reported in the available databases (8.2% (*BRCA1*) and 11.5% (*BRCA2*)). These NPR variants may possibly represent unique variants in our population, or they are simply not reported to international databases from other laboratories yet.

The amount of rare mutations found in our study may be similar in other countries assumed to have a strong founder effect. In a recent study from Bulgaria, 200 individuals from breast/ovarian cancer families were genotyped with sequencing, and comparable results were found [24]. Two new, and five previously known mutations were identified in *BRCA2*, while two new and six previously known mutations were identified in *BRCA1*. In a Danish study on *BRCA1/2* founder mutations, a majority of the mutations identified were found one individual or family, which is similar to our study [25]. A rate of 7–13% rare, non-founder mutations has been described in Ashkenazi-Jewish *BRCA1/2* carriers [26]. In the Polish population, 10 % of breast/ovarian cancer patients that previously tested negatively for the Polish founder mutations were found to carry other recurrent or founder candidate variants [27]. The questions following from this are what should the indication for *BRCA1/2* testing be, and which method for testing will have sufficient sensitivity. Founder mutation testing alone will, according to this, even in founder populations have lower sensitivity than favourable.

To establish the frequencies of rare pathogenic *BRCA1/2* mutations is very important due to their significance in cancer prevention [28], also when a broader testing approach for other breast cancer genes with lower penetrance is applied through gene panels [26, 29]. Founder mutations and their effect will dilute in a multi-cultural society as described in this study. If presymptomatic population screening should be discussed in Norway, as it has been piloted for Ashkenazi Jews in the United States, such knowledge is nevertheless crucial [30]. When discussing screening for any disease, rare or common, establishing test sensitivity and specificity is central [31]. If a similar offer of voluntary founder testing in subgroups of the Norwegian society should be planned for, these data can be used to establish the expected false negative rate. On the other hand, if sequencing/MLPA is considered a better choice because of higher detection rate of pathogenic variants, the rate of detecting VUS and the practice of reporting these variants and reevaluation over time must be considered. To establish the frequency of VUS in the patient database was outside the scope of this study, but after the conversion by the laboratory to full gene tests by sequencing combined with MLPA in January 2014, the rate of VUS in diagnostic testing of *BRCA1/2* has been 4.9% [32].

Knowing the local mutation spectrum also makes it possible to plan for future epidemiological studies in the larger population, haplotype studies and possibly genotype/phenotype studies. Norwegian founder mutations have previously been considered to have somewhat lower penetrance and lower cancer risk per year than the rarer mutations in the population regarding both breast and ovarian cancer [33]. The issue of possible genotype-phenotype effects in *BRCA1/2* mutation carriers have been explored in several studies [22, 34, 35]. Rebbeck et al. presented in 2015 one of the largest studies performed on the subject, confirming the existence of areas with relative variation in breast and ovarian cancer risk. The results await appropriate validation before findings may be transferred to clinical counselling practice.

There are some selection biases due to the changing practice of both patient inclusion and testing over the years. During the first 10 years of *BRCA* testing in OUH, patients were mainly tested for the known founder mutations. Family members of identified mutation carriers were informed by their relatives of the possibility of predictive testing, and over time, quite large families with founder mutations were identified. An overall larger percentage of carriers than of families for the most frequent mutations illustrate this, as well as the rate of mutation carriers per family (stated in Tables 2 and 3). The rarer mutations in *BRCA1* have a larger fraction of families compared to the fraction of mutation carriers, and therefore a lower number of mutation carriers per identified family. The high number of different families per founder mutation may however indicate that this family testing strategy is not the sole reason for the high variant frequency, but rather confirm what is known about these mutations already. The variants are old, present before the historical event of the Bubonic plague in fourteenth Century. The carriers have been the object of high selection i.e. through bottle neck phenomenon, non-random mating/inbreeding, as well as other historical factors favouring establishing large families [13, 16]. Over time the families have grown so large that the descendants loose contact, and hence the number of seemingly unrelated families increase. For *BRCA2*, the average number of mutation carriers per family is quite similar between the frequent and the rare mutations. This may be due to a true, but weaker founder effect for the most frequent *BRCA2* variant, c.5217_5223del, i.e. a younger age of this variant than the *BRCA1* founder variants, and hence a true, lower frequency in the population. This may in turn be caused by less selection favouring the mutation, e.g. lower degree of inbreeding, smaller families and other historical factors. However, the numbers may also simply reflect a shorter time span both since the most frequent mutation, c.5217_5223del, was identified in our clinic.

Defining a mutation as rare when identified in two families, and as “less frequent” when found in three families or individuals may seem a bit arbitrary, and even misleading. As shown in Tables 2 and 3, variants found in i.e. three small families consisting of 1–2 persons each may really also represent a rare mutation, and if counted as such it would lead to a higher number of rare mutation carriers. The material represents more than half of the Norwegian population, but are not representative for the nation as such. There are well-known local, founder effects present in both the Western and Northern part of the country that will influence on the national frequencies of founder mutations. Lower inclusion rate of patients especially from Western Norway in the later years may also bias the result presented here towards a lower proportion of these mutations in our patient cohort.

In sum, we find that while the well-known founder effect in Norway is still present, it is weaker than previously described. Several frequent mutations detected over the last 15 years are considered founder candidates, and previously described founders from other populations are also found among rare variants in our population. Due to the significant presence of rare mutations we suggest that in order to identify as many *BRCA1/2* mutation positive families as possible one should consider to systematically offer retesting with sequencing and MLPA to individuals and families that have previously only been tested with a limited, founder mutation test. The study also supports the continuation of the introduced testing practice of using sequencing and MLPA as initial test in individuals fulfilling testing criteria. Such a testing practice will over time allow detection of variants, both rare and frequent, that otherwise would be missed. Cost-efficiency of such a test approach will vary among health care systems. However, a similar practice has been shown to be cost- efficient in a recent UK study, especially when allowing healthy mutation positive relatives to be identified before they contract cancer [36].

## Conclusions

The mutation spectrum of *BRCA1* and *BRCA2* mutations in the largest hereditary cancer clinic in Norway is diverse. The four *BRCA1* founder mutations identified in 2001, are still the most frequent *BRCA1* mutations, but account now for 52% of BRCA1 mutation carriers, compared to 68% in 2001. In total, 46 % of the registered *BRCA1/2* families (454/981) had one of the previously known Norwegian founder mutations, identified through the founder search in Alamut. Moreover, several frequent mutations have been identified during the last 15 years, many of which are considered founders in the Norwegian population. Lastly, a majority of mutations are rare, but as a group these rare mutations account for 15% of *BRCA1* and 25% of *BRCA2* mutation families. The results presented therefore support the current practice of using sequencing and MLPA over limited testing for only founder mutation in our patient population. Only through this strategy will new *BRCA1/2* mutations, both rare and frequent be identified. Families and individuals who previously have tested negative for founder mutations should systematically be offered retesting with sequencing and MLPA in order to identify healthy *BRCA1/2* carriers and enable them to prevent cancer.

## Abbreviations

ACMG: American College of Medical Genetics
*BRCA1/2*: Breast cancer gene 1 / 2
BRCT-domain: *BRCA1* - C terminus domain
ClinVar: Public Archive of variants, hosted by National Center of Biotechnology Information
ENIGMA: Evidence-based Network for the Interpretation of Germline Mutant Alleles
F: Founder mutation
HGMD: Human gene mutation database
HGVS: Human genome variation society
HTS: High throughput sequencing
LOVD: Leiden open (Source) variation database
MLPA: Multiplex ligation-dependent probe amplification
MRI: Magnetic resonance imaging
NF: Not founder mutation
NPR: Not previously reported
OUH: Oslo University Hospital
RING-finger domain: Really interesting new gene - finger domain
UMD/BRCAShare: Universal mutation database - BRCA
VUS: Variants of unknown significance
